# Cancer Survivors’ Receptiveness to Digital Technology–Supported Physical Rehabilitation and the Implications for Design: Qualitative Study

**DOI:** 10.2196/15335

**Published:** 2020-08-05

**Authors:** Sine Rossen, Lars Kayser, Jette Vibe-Petersen, Jesper Frank Christensen, Mathias Ried-Larsen

**Affiliations:** 1 Centre for Physical Activity Research Rigshospitalet Copenhagen Denmark; 2 Copenhagen Centre for Cancer and Health Municipality of Copenhagen Copenhagen Denmark; 3 Department of Public Health University of Copenhagen Copenhagen Denmark

**Keywords:** cancer, rehabilitation, physical activity, digital technology

## Abstract

**Background:**

Physical activity is associated with a positive prognosis in cancer survivors and may decrease the risk of adverse effects of treatment. Accordingly, physical activity programs are recommended as a part of cancer rehabilitation services. Digital technology may support cancer survivors in increasing their level of physical activity and increase the reach or efficiency of cancer rehabilitation services, yet it also comes with a range of challenges.

**Objective:**

The aim of this qualitative study was to explore cancer survivors’ receptiveness to using digital technology as a mode of support to increase their physical activity in a municipality-based cancer rehabilitation setting.

**Methods:**

Semistructured interviews were conducted with 11 cancer survivors (3 males, 8 females, age range 32-82 years) who were referred for cancer rehabilitation and had participated in a questionnaire survey using the Readiness and Enablement Index for Health Technology (READHY) questionnaire. Data analysis was based on the content analysis method.

**Results:**

Two themes were identified as important for the interviewees’ receptiveness to using digital technology services in connection with their physical activity during rehabilitation: their attitude toward physical activity and their attitude toward digital technology–assisted physical activity. Our results indicated that it is important to address the cancer survivors’ motivation for using technology for physical activity and their individual preferences in terms of the following: (1) incidental or structured (eg, cardiovascular and strength exercises or disease-specific rehabilitative exercises) physical activity; (2) social or individual context; and (3) instruction (know-how) or information (know-why).

**Conclusions:**

The identified preferences provide new insight that complements the cancer survivors’ readiness level and can likely help designers, service providers, and caregivers provide solutions that increase patient receptiveness toward technology-assisted physical activity. Combining digital technology informed by cancer survivors’ needs, preferences, and readiness with the capacity building of the workforce can aid in tailoring digital solutions to suit not only individuals who are receptive to using such technologies but also those reluctant to do so.

## Introduction

Increased physical activity among cancer survivors is associated with increased survival and a lower risk of cancer recurrence, particularly among breast and colorectal cancer survivors [1-5]. Physical activity is also associated with decreased cancer-related fatigue and sleep disturbances [6], increased health-related quality of life [7], and decreased treatment-related adverse effects [5,8]. The general recommendation for adults is at least 150 minutes per week of moderate-intensity physical activity [9]. Accordingly, the incorporation of physical activity is recommended in cancer rehabilitation services [10,11]. Participating in rehabilitation is a challenge for some cancer survivors. In the United States, up to 42% of cancer survivors do not meet the recommendations for physical activity [12]. In Copenhagen, Denmark, only 48% of those referred to municipality-based rehabilitation participate in the group-based strength and cardiovascular exercise that is available. Studies have shown that reasons for not participating in exercise interventions can be both cancer-specific (ie, symptoms such as fatigue and pain) and situational/environmental (eg, distance to exercise facilities, time of day classes are held, or other commitments) [13,14].

It is estimated that the number of cancer survivors in need of cancer rehabilitation will increase in the coming years due to increasing cancer incidence and higher survival rates [15,16]. The increasing prevalence of cancer survivors and scarce resources (ie, increasing costs and declining health professional workforce) challenge rehabilitative services, for instance, in the form of longer waiting lists and shorter appointment times per patient for in-person services [17].

Digital technology can perhaps help cancer survivors increase their level of physical activity as technology can serve to resolve the geographical and logistical obstacles associated with traditional programs requiring in-person supervision and communication, increasing the efficiency of rehabilitative services [18-20]. Examples of digital technology interventions are applications offered via smartphones and websites, as well as wearables that can be used to instruct, monitor, or motivate physical activity [21-27]. When introducing digital services, it may be important that the provider or service organization understands how and to what extent the digital technology may be beneficial for the individual. Such a stratification will play an important role in requirement specifications for digital solution providers [28]. In addition, when identifying and excluding people unable to take advantage of technology, it may be possible to allocate additional resources for in-person supported services for this group.

**Figure 1:**
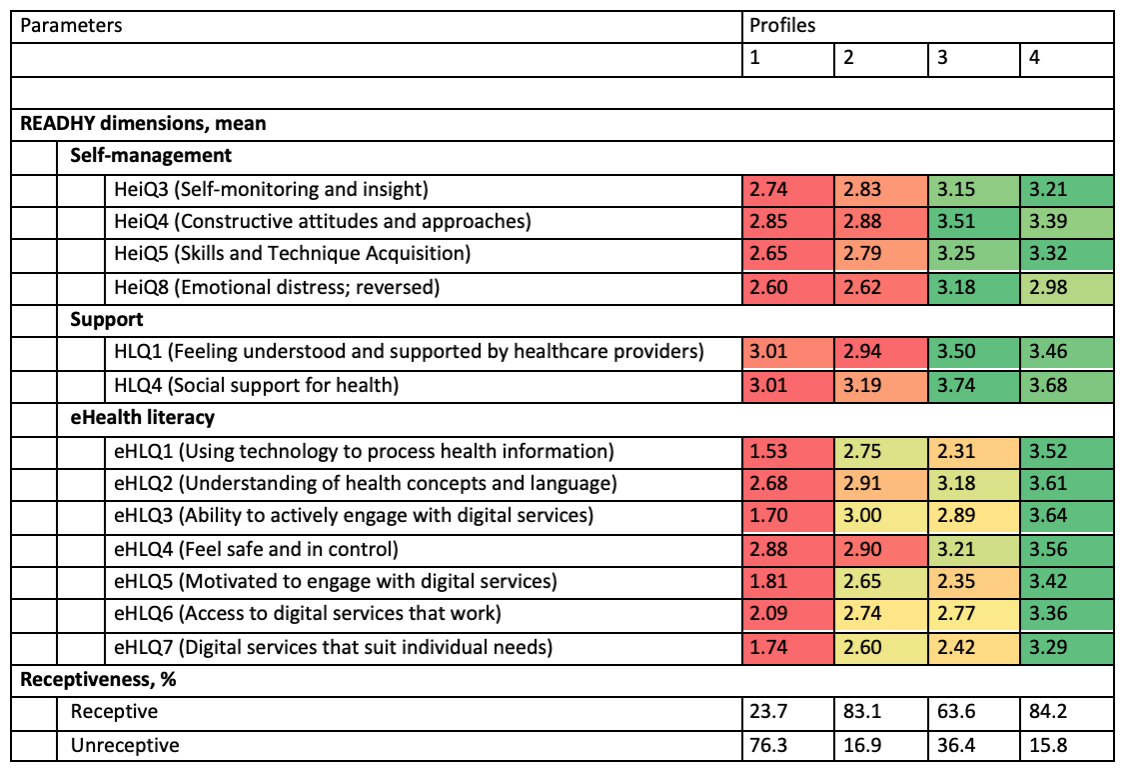
Four health technology readiness profiles and their receptiveness to supplement physical activity during rehabilitation with technology. The READHY scale ranges from 1 (Strongly disagree) to 4 (Strongly agree). Within each dimension, the average READHY scores are color coded relative to the other profiles from red (lowest score) to green (highest score). Adapted from [29,34]. eHLQ: eHealth Literacy Questionnaire; HeiQ: Health Education Impact Questionnaire; HLQ: Health Literacy Questionnaire; READHY: Readiness and Enablement Index for Health Technology.

We previously reported how cancer survivors referred to rehabilitation can be stratified into four distinct profiles according to their health technology readiness using the Readiness and Enablement Index for Health Technology (READHY) [29], an instrument based on the eHealth Literacy Framework [30] and the eHealth Literacy Questionnaire [31], supplemented by the social dimensions of the Health Literacy Questionnaire [32] and by the self-management dimensions of the Health Education Impact Questionnaire [33]. The dimensions relating to electronic health (eHealth) literacy described user knowledge and skills, the intersection between users and technologies, and users’ experience of systems. The dimensions relating to self-management add knowledge about the individuals’ ability to handle their condition and emotional response. The social dimensions add knowledge about the individuals’ social context (ie, support from family and friends or health professionals) [29]. The four identified READHY profiles (Figure 1) differ regarding their receptiveness to physical activity rehabilitation supplemented by digital technology. In this context, receptiveness is based on whether the interviewees can imagine supplementing physical exercise with technology (eg, a smartphone, computer, or smartwatch). This means that receptiveness not only addresses their technology readiness but also their intention to perform or their attitude toward physical activity [34].

The quantitative approach provided important information about the four different profiles and their level of readiness for technology. The stratification of users can serve designers and service providers in addressing the various profiles of service users in relation to their overall characteristics. However, patient receptiveness to technology-supported physical activity cannot be explained solely by their technology readiness level. Other aspects, such as the cancer survivors’ underlying assumptions and reasoning, may facilitate or constitute a barrier to usage of health technology [35], and should be more thoroughly investigated. Therefore, the purpose of this study is to enhance knowledge on receptiveness to technology-assisted physical activity in cancer survivors and to give a broad spectrum of cancer survivors a voice by taking their various levels of readiness into consideration. Consequently, our research questions are the following:

1. What are the cancer survivors’ assumptions and reasoning regarding health technology in relation to physical activity?
2. How may these assumptions influence their receptiveness to using digital technology in relation to their physical activity during rehabilitation?
3. Do needs and preferences vary between the profiles identified?

A qualitative approach can provide a more nuanced description of the profiles [36], enabling us to identify differences that can be used to tailor services and identify those who may not benefit from technology in physical activity rehabilitation programs.

## Methods

The methods section is reported according to the Consolidated Criteria for Reporting Qualitative Research (COREQ) [37].

### Setting

This study took place at the Copenhagen Centre for Cancer and Health in Copenhagen, Denmark. In 2007, the main responsibility for the rehabilitation of patients with cancer in Denmark was transferred from the regional level (hospital management) to the municipal level, in accordance with the current trend of offering more people-centered services [38]. The Copenhagen Centre for Cancer and Health provides interdisciplinary rehabilitation of patients with cancer that includes group-based strength and cardiovascular training and individually tailored rehabilitative exercises and health education, as well as individual counseling in relation to health problems, diet, occupation, and economic issues. Since cancer survivors can be referred to rehabilitation in their initial treatment period, many of them are undergoing active treatment while participating in rehabilitation programs.

### Study Design and Interview Participants

This qualitative study is part of a large cross-sectional study exploring health technology readiness in 305 patients with cancer referred to public rehabilitation services at the Copenhagen Centre for Cancer and Health [29,34]. Briefly, participants did the READHY questionnaire measuring self-management, social support, and eHealth literacy, in addition to filling out a background information questionnaire. Subsequent cluster analysis identified four cluster profiles differing regarding their health technology readiness profiles and sociodemographic variables, such as age, education, number of chronic conditions, and receptiveness to technology in relation to physical activity [29,34]. Our goal was to invite three participants from each of the four profiles for interviews. Potential interviewees were purposely selected for interviews by the first author, SR (female), based on their READHY profile, sex, and age, without any other characteristics taken into consideration, including diagnosis. In total, 23 (12 females, 11 males) of the 305 patients were invited by mail and then contacted by phone after 7 days by a staff member at the Copenhagen Centre for Cancer and Health to inquire about participation. Those who agreed to participate (n=11, 8 females, 3 males) were contacted by SR to make an interview appointment. Of those who did not participate (n=12), 5 could not be reached after 3 attempts by phone, and 7 declined participation. Reasons for declining were not requested. Interviewees received oral and written information about the study and gave written informed consent before being interviewed. Interviewees were interviewed between June 2017 and October 2017 by SR. Interviews were conducted in an interview room at the center (n=9) or at the interviewee’s own home (n=2). No one else was present during the interviews, and interviewees were not compensated for their participation. The interviewer knew which READHY profile the interviewee belonged to but focused on keeping an open mind during the interviews. No repeat interviews were conducted. The interviewer is a postdoctoral fellow at the Centre for Physical Activity Research at Rigshospitalet, University of Copenhagen Hospital, and was supervised by author LK from the Department of Public Health, University of Copenhagen. The semistructured interview guide with prompts was designed to enable a deeper exploration of our previous findings and to provide us with insight into the cancer survivors’ underlying assumptions and reasonings regarding their receptiveness to using technology in relation to physical activity during rehabilitation. As a result, we based the questions on the READHY questionnaire addressing self-management, including the impact of their condition; social support from health professionals and their personal network; and their eHealth literacy. We also included questions on exercise/physical activity and digital technology–assisted physical activity (Multimedia Appendix 1). A digital audio recording of each interview, which lasted 15 to 45 minutes (mean 28 minutes), was made and transcribed verbatim.

### Data Analysis

Interviews were analyzed using directed content analysis [39,40]. All interview transcripts were read and reread by SR to ensure familiarity with the data. We used an abductive approach [40]. The first step was deductive based on the READHY framework; a codebook [41] was constructed based on three concepts in the READHY framework (self-management, support, and eHealth literacy) and receptiveness to technology-assisted physical activity (Multimedia Appendix 2). SR then marked all the passages in the transcripts that appeared to relate to the codes. NVivo 12 (QSR International) was used to organize and code the data. After coding, SR and LK identified two themes. In the second step, which was inductive, SR and LK revisited the text and codes to identify subcategories within the themes and achieved consensus using abstraction and interpretation processes [40]. In the abstraction process, it became evident that when discussing physical activity in connection with rehabilitation, interviewees did not distinguish between exercise and specialized disease-specific exercises. For the interpretation, the category physical activity was revisited by SR using the following: (1) the term “rehabilitative exercise” for specialized cancer-specific exercises to alleviate disease or treatment-related problems (eg, mobilization of scar tissue, self-managed manual lymph drainage, and swallowing exercises for dysphagia) and (2) Caspersen and colleagues’ [42] definition of physical activity (“bodily movement produced by skeletal muscles that results in energy expenditure”), which can be further classified as incidental or structured. Structured physical activity or exercise is a subset of physical activity that is planned, structured, and repetitive, with the purpose of improving fitness or health [42,43]. Incidental physical activity is unstructured activity that is part of daily living at work or at home, such as walking or cycling for transport, climbing stairs, and doing housework [44]. SR and LK selected the quotations presented in this study.

### Ethics

According to Danish law, formal ethical approval was not required because no biological material was obtained in the study. The study was conducted in accordance with the Helsinki Declaration and approved by the Danish Data Protection Agency (2015-55-0630). All participants were informed about the study before starting the interviews, received a participant information sheet, and were informed that their participation was voluntary, that they were ensured anonymity, and that all data would be handled confidentially. Written informed consent was obtained.

## Results

### Overview

In total, 8 women and 3 men were interviewed; they ranged from 32 to 82 years of age and represented all four READHY profiles (Table 1). Based on their statements, the interviewees were further characterized as being users (or nonusers) of digital technology for physical activity if they stated that they used or had used (or not) smartphone apps, websites, or wearables for physical activity. Interviewees generally owned technological devices, eg, smartphones, tablets, or computers, using them for various everyday purposes. Of the 11 interviewees, 5 were receptive to using technology for physical activity during rehabilitation. In total, 5 of the 11 participated in strength and cardiovascular exercise at the center (75 minutes twice weekly for 16 weeks). The interviewees represented 8 different International Classification of Diseases Version 10 cancer sites: lip, oral cavity, and pharynx (C00-C14; n=1); digestive organs (C15-C26; n=2); respiratory and intrathoracic organs (C30-C39; n=1); breast (C50; n=3); urinary tract (C64-C68; n=1); eye, brain, and other parts of central nervous system (C69-C72; n=1); lymphoid, hematopoietic, and related tissue (C81-C96; n=1), and ill-defined, secondary, and unspecified site (C76-C80; n=1). At the time of the interview, 2 participants were still undergoing active treatment. Most of the noninterviewees were male (8 males, 4 females). They ranged in age from 28 to 70 years (mean 56 years), and generally owned smartphones, tablets, or computers, using them for various everyday purposes (Multimedia Appendix 3).

**Table 1:** Characteristics of interview participants.

ID	Sex	Age	READHY profile^a^	Physical activity digital technology usage^b^	Receptive^c^	Technology ownership^c,d^			Usage^c^		Purpose of using technology^c,e^					
						S	T	C				W	IS	Cm	P	E
1	F	82	1	Nonuser	No			✓		Several times daily					✓	
2	F	69	1	Nonuser	No	✓				Several times daily				✓		
3	F	43	2	Nonuser	Yes	✓	✓	✓		Several times daily		✓	✓	✓	✓	
4	F	71	2	User	Yes	✓	✓	✓		Several times daily			✓		✓	✓
5	F	65	3	User	No	✓	✓	✓		Several times daily		✓	✓	✓	✓	
6	M	32	3	User	Yes	✓	✓	✓		Several times daily		✓	✓	✓		✓
7	F	63	3	Nonuser	No	✓	✓	✓		Once per day			✓	✓		
8	F	66	3	User	Yes	✓	✓	✓		Several times daily			✓	✓	✓	✓
9	M	64	4	Nonuser	No	✓	✓	✓		Several times daily		✓	✓	✓	✓	
10	M	36	4	User	Yes	✓		✓		Several times daily		✓	✓	✓	✓	
11	F	67	4	Nonuser	No		✓	✓		A few times per week				✓		

^a^READHY: Readiness and Enablement Index for Health Technology.

^b^This is based on the interviewees’ statements.

^c^These are based on questionnaire data reported in [34].

^d^S: smartphone; T: tablet; C: computer.

^e^W: work; IS: information seeking; Cm: communication; P: practicality; E: exercise.

During the content analysis, we identified four subcategories as important to understanding the interviewees’ assumptions and reasonings in relation to digital technology-assisted rehabilitation, divided into the following two themes: (1) attitude toward physical activity within the subcategories incidental and structured physical activity and social relations (ie, with other cancer survivors, exercise participants, or health professionals when performing physical activity); and (2) attitude toward technology-assisted physical activity within the subcategories motivation and prerequisites. Table 2 provides a sample of quotes illustrating the two themes and related subcategories.

**Table 2:** Quotes illustrating the “attitude toward physical activity” and “attitude toward technology-assisted physical activity” themes.

Attitudes and subcategories		Profile 1 quotes	Profile 2 quotes	Profile 3 quotes	Profile 4 quotes
**Attitude toward physical activity**					
	**Incidental or structured (exercise) physical activity**				
		*I’ve never done that [exercised]. Well, I did gymnastics back in the day. I’ve done sports, right? I’ve skied a lot. Slalom, up and down white mountains, that sort of stuff, you know? I don’t do that anymore, but I haven’t had the need. You didn’t do that when I was a kid. You didn’t exercise, you climbed trees and stuff, you know?* [Female, profile 1, ID1] *It can be hard to motivate yourself to exercise in this [cancer disease], and what you’re going through with the disease and stuff. So, it’s fine [exercising at the center], and it got me started exercising. I’m quite happy about that, even though I think every time, “Oh no,” you know? But you’re quite contented when you leave and think “That’s probably why I feel fine.” Because, as I said to you, I get chemo and I don’t feel any side effects. […] I don’t want to go to a fitness center.* [Female, profile 1, ID2]	*I’ve been extended for two more months [team-based exercise at the center] because they know if they let me go, I won’t exercise. […]. Because, I'm sorry to say, I don’t like to do exercise. […] I think it hurts. I think it's hard. I hate sweating.* [Female, profile 2, ID3] *I was in a really good physical shape before […] I’m good at riding my bike to go for a swim in the morning [at the beach]; I do winter swimming, and I’m active.* [Female, profile 2, ID4]	*I dance at a fairly high level and do yoga on a fairly high level.* [Female, profile 3, ID5] *I feel agile enough, but, but, I should, you know [do regular exercise]? And I don’t like those centers at all [fitness centers]. […] I felt so happy about coming here [rehabilitation center], so it wasn’t a problem getting up in the morning in the middle of the winter and biking here twice a week. […] I haven’t done much exercise. I really haven’t attended anything other than yoga. No hard physical exercise. I’ve just rushed around in my everyday life, you see?* [Female, profile 3, ID7] *I used to work out every other day before. Strength exercises. […] [The physiotherapist] gave me some exercises I can do at home and I have an app called 7-minute workout. That actually works really well to get a little exercise. Biking to work, going for long walks*. [Male, profile 3, ID6] *For the past 30 years I’ve exercised my left side to keep it going [rehabilitative exercises]. And I just continued that on the right side. Little exercises at first and gradually more, you know? […] Even on days when you think you haven’t walked much, and we’ve just been at home. We’ve walked 6-7,000 steps anyway, you know? Well, we have a little yard. I take care of it myself, and there are a lot of stairs.* [Female, profile 3, ID8]	*I haven’t been able to do badminton and tennis like I used to. But I’ve walked or biked to work. […] At work we have exercise facilities. So, I could go there after work or during my lunch break or whatever suited me. […] In the beginning you were sick. You had to do it. In some way, doing it was more legitimate. Now I’m not sick; then there’s so much other stuff.* [Male, profile 4, ID10] *I don’t want to do exercise [in a gym] in the summer when I have a small plot of land. I do plenty in the yard, mowing the lawn, cutting hedges, biking back and forth, and other stuff. I have a deal with the fitness center that I take the summer months off. And then from October to May I do exercise [in the fitness center].* [Female, profile 4, ID11] *I went to a fitness center before, and I continued after the operation. And used the exercises I got from here [the rehabilitation center]. […] In the summer, I don’t go [to the fitness center]. I bike, and I play golf and other stuff. I think I get enough exercise. But, in October, I start [going to the fitness center].* [Male (profile 4, ID9]
	**Social relations**				
		*When you’re walking, of course you have to be aware of not just trudging along, but I usually think it’s cozier walking with someone.* [Female, profile 1, ID2]	*[…] If it's on my calendar, if I had an appointment with you. Well, then I would do it. It could also just be a personal trainer who leads me through the first program. Just to say, “Well okay, she's shown up.” Just like you do [rehabilitation center]. Then I would go.* [Female, profile 2, ID3] *[…] I can’t see myself sitting at home doing exercise, I’m too social for that. […] I appreciate the other women who I exercise with.* [Female, profile 2, ID4]	*[…] there’s a sense of safety in the almost family-like atmosphere when you go through the door. […] You see people’s hair grow, and they get color in their cheeks, and you see someone who is dragging themselves along, and you think, “I’m glad that’s not how I’m feeling anymore.” […] When you sweat together, then you have something to share. And you have a little chat afterwards over a cup of coffee.* [Female, profile 3, ID7] *For me, it’s important that we’re doing it together [exercising].* [Female profile 3, ID5]	
**Attitude toward technology-assisted physical activity**					
	**Motivation**				
		*If anyone paces me, tells me what to do, I get annoyed and obstinate. I’ll decide that myself. And I’ll do it in my own pace. Well, I would say no thanks. But I haven’t tried. But I could imagine that was how I would react. Yes, yes [laughs]. [...] I can just say, “Okay, I can delete the app.” Or I can say, “Hey, nobody’s there.” So, it probably has to be more personal, if I have to do it.* [Female, profile 2, ID3] *It’s hypothetical, right? Because, again, will I actually do it? [use, eg, an app to exercise].* [Female, profile 2, ID4]	*I thought, “that might be fun.” The other day, I was walking with my daughter and she said to me, “Mom, we’ve walked 4.6 miles.” “Super,” I replied. You can see that on the, what’s it called, the GPS thing. […] Because it tells you how far you’ve walked and stuff, you know? And I think that’s clever. But stuff like my heartrate and all that. I don’t want that.* [Female, profile 1, ID2]	*Deep down, I want to use it because I can see that it’s convenient for many people. But you wouldn’t get the social dimension that you get in this building, you know? [rehabilitation center]. […] I have a hard time finding a place I want to go and continue my exercise. If I knew, I would spend half an hour and turn on my phone every morning. Why don’t I do that? Then, I wouldn’t have to do it at five pm, in the rain, in November, but in the morning when it suits me the best.* [Female profile 3, ID7] *I don’t think I would want to do that at all [use an app for exercise]. […] My attitude towards using my body is that it has to be fun. And, it has to be entertaining, and it has to be nice. For me, I’m not interested in how high my pulse gets. Or how many pounds I lift. [..] I don’t think an app should decide how I should move. […]* [Female, profile 3, ID5]	*If it works [an app], I might consider using it. But I don’t know what I should use it for [because she keeps herself physically active all the time]. [Female, profile 4, ID11]* *I would ask the instructor [fitness instructor], “Would you do a training/exercise program for me?” I haven’t thought about using an app. […] It might be a good idea. I just haven’t thought about it. I always run around with a piece of paper in my bag with my exercises on it.* [Male, profile 4, ID9]
	**Prerequisites**				
			*Well, it should be what I’m writing on this paper now. Date, how many pounds, and how many times I’ve lifted, and stuff like that. […] There’s this fantastic game. It’s a mental exercise app. And there’s this curve that shows that I didn’t do well today. It motivates me by saying at 9 am, “It’s time to do your games.” And I think that's exciting. And I can see that something’s happening, and I can see when it isn’t. Then it's a bit fun, and I can also see if I'm progressing.* [Female, profile 2, ID3]	*I think, if it were something I could do at home, some sort of morning program. I know someone who has an aerobic step in front of their tv, you know? And then they get it done in the morning. It doesn’t matter what you do. I would easily have the time to do that for half an hour in the morning.* [Female profile 3, ID 7] *If I had to use an app, then maybe it could be a yoga app […] Yoga is a guided activity, and you can do it yourself, but you have to know exactly how to combine them [the exercises] if you want to do something you’re not used to.* [Female, profile 3, ID5] *You should be able to adjust the settings to, for example, I want to do it in the morning, at noon, and in the evening, like I do now. And then it goes “beep, beep,” remember your exercises [rehabilitative exercises]. And you could bring it to work. It’s always in your pocket, you see? […] It could be smart if you could call or send a message to your main contact person [at the rehabilitation center]. […] It’s good exercise, where you get support and you have like a coach saying to do ten push-ups. The app [7-minute workout] actually says, “Do ten push-ups.” Then you can put it on your bookcase, clear some space in the* *living room, and do all the exercises. That works really well. And you can do it at 5:30 am or 3:45 pm.* [Male, profile 3, ID6] *Maybe more enthusiasm. It’s hard to explain, but one thing is that you’re shown the exercises. But I also need to know why. […] Why is this exercise better than another one […] A little more depth. And I think technology would be good at/for that.* [Female, profile 3, ID8]	*It should contain instructions on the exercises I should do. I mean, how they look. Describe how they look. So that you can see what you’re supposed to do, you know? […] That would make sense to me. That would be clever, because I already use my phone to listen to music when I exercise. Then I could just check, “What’s the next exercise?” That would be fantastic.* [Male, profile 4, ID9] *These exercises are especially good; you should do them like this and this.” I mean, how much you should do or how hard it should be, but it should make sense to you. It has to be something where I’ve been asked or assessed, “This is important for you.” […] If there are three days a week where I should do it, what happens if I skip a day? Should I do more then? […] Where you understand what the different exercises [rehabilitative exercises] do, and if you don’t’ do them, what should you do instead, and what are the consequences? […] It could also be fun to record my weight, repetitions, how much, and then see if there is any progress.* [Male, profile 4, ID10]

### Attitude Toward Physical Activity

The theme “attitude toward physical activity” is related to what the participants think about physical activity and how the participants prefer to be physically active (ie, incidental or structured physical activity, in a social context or individually).

#### Incidental and Structured Physical Activity

Across all profiles, 8 interviewees talked about their incidental physical activity in relation to both transportation (eg, bicycling) and leisure time (eg, gardening, using the stairs). They considered these activities as a supplement or a substitute for exercise (structured physical activity). In profiles 1 and 2, (low self-management, low to medium eHealth literacy), 3 of 4 interviewees were reluctant to participate in organized physical activity, whereas the fourth was accustomed to organized team-based exercise (fitness center, yoga), emphasizing the social aspect of structured physical activity. In profiles 3 and 4 (high self-management, medium to high eHealth literacy), 5 of 7 interviewees were accustomed to participating in structured physical activity. All 3 interviewees in profile 4 had performed structured physical activity regularly before the cancer and complementary to the services offered by the rehabilitation center. In addition, two of the profile 4 interviewees mentioned that the time of year affected their activity level; they did not use structured physical activity facilities in the summer because they were generally more active in their everyday lives.

#### Social Relations

This context refers to social relationships with other cancer survivors or exercise participants and health professionals or trainers in connection with performing physical activity. There was a tendency among the individuals in profiles 1 and 2 to mention social relations as important. For example, 3 of 4 interviewees in profiles 1 and 2 (low self-management, low to medium eHealth literacy), and one interviewee in profile 3, specifically mentioned social relations as important (either social relationships with other exercise participants, social relationships with other cancer survivors, or coaching from staff and being accountable to others). None of the other interviewees mentioned the social aspect of physical activity as important to them.

### Attitude Toward Technology-Assisted Physical Activity

The theme “attitude toward technology-assisted physical activity” is related to the interviewees’ motivation to use technology for physical activity and the prerequisites they have if they were to use technology for physical activity. One interviewee (ID1, low self-management, low eHealth literacy) was not open to the idea of exercising and, as such, could not relate to rehabilitation assisted by digital technology. Among the remaining 10 interviewees, none of them clearly rejected digital technology–assisted physical activity.

#### Motivation

The participants’ motivation for using digital technology (eg, an app or website) in connection with physical activity ranged from being open-minded to being reluctant. For example, 2 interviewees (profile 1, low self-management and eHealth literacy; profile 4, high self-management and eHealth literacy) were nonusers of technology in connection with physical activity and had never thought about using it but were open to the idea. They saw the potential benefits of using it, such as being aware of the miles walked and not having to run around with a piece of paper describing the exercises. However, 5 interviewees (profiles 2 and 3, medium self-management and eHealth literacy; profile 4, high self-management and eHealth literacy) were reluctant to use digital technology in connection with structured physical activity because they would miss the social aspect of exercising, were active already, and could not see the purpose of an app or website; they did not like to be told what to do by, for example, an app, and held the assumption that the role of technology is to provide data about activity rather than to contribute to a good experience. However, 3 individuals that were reluctant to use digital technology already used or had used digital technology for physical activity (eg, to count steps using their phone’s built-in pedometer or to watch online exercise or rehabilitative exercise instruction videos).

#### Prerequisites

The subcategory “prerequisites” includes the interviewees’ preferences for instruction, information, and setting in relation to the use of technology for physical activity. Among participants, 7 interviewees, mainly from profiles 3 and 4 (high self-management, medium to high eHealth literacy), and mainly users of digital technology for physical activity (both structured and incidental) had preferences about what they would like digital technology to do, if they were to use it during rehabilitation. Some interviewees focused on technology for their rehabilitative exercises, with the aim of improving or maintaining a specific function (eg, being reminded of swallowing exercises or memory exercises, and instructions on how to perform rehabilitative exercises to regain function after an operation). Others talked about technology as a support for structured physical activity, like yoga or cardiovascular exercises, either to be used in their own home or in a fitness center. In general, profiles 3 and 4 (high self-management, medium to high eHealth literacy) expressed a need for support to make decisions in connection with training using technology. They wanted to know why they should do physical activity and what the benefits of exercise are. For example, 2 interviewees said that digital technology could provide information about why they should do exercise, not just how to exercise, and one interviewee believed this might increase her enthusiasm for exercising. One interviewee mentioned the relevance of a yoga app because yoga demands instruction and knowledge of how to combine postures. In addition, 2 interviewees mentioned the availability of visual instructions to be able to see how the various exercises are performed. One interviewee thought that technology may contribute to devising an individualized plan based on an assessment of his condition and this plan could be adapted to day-to-day changes in activity levels. The convenience of being able to exercise on demand when using technology and monitoring progression was also mentioned. One interviewee mentioned reminders of when to do rehabilitative exercises and the possibility of getting in contact with the center contact person/health professional directly through an app.

## Discussion

### Principal Findings

In this study, we give cancer survivors a voice in a rehabilitation setting to understand their assumptions and reasonings about digital technology–assisted physical activity in general, and its possible implications for the future design and introduction of technology. Recruiting individuals from each of the four health technology readiness profiles ensured that perspectives from a wide range of cancer survivors were included. We identified two important themes in terms of cancer survivors’ receptiveness to using digital technology in relation to their physical activity during rehabilitation: (1) attitude toward physical activity, with the subcategories “incidental and structured physical activity” and “social relations,” and (2) attitude toward technology-assisted physical activity, with the subcategories “motivation” and “prerequisites.” The two themes and related subcategories describe the thinking of the interviewees and contribute to an understanding of how interventions can be designed to address both attitude toward exercise and attitude toward technology. Our previous quantitative data [34] suggests that not everyone is receptive to and able to use digital technologies to increase and maintain physical activity in connection with rehabilitation. This is supported by our interviewees. The results indicate that it is important to address the cancer survivors’ motivation (ie, the degree to which they are open-minded about using technology for physical activity). Another aspect of motivation is how to include the cancer survivors’ particular needs, such as their preferences, in relation to physical activity, in terms of the following: (1) incidental or structured physical activity (where structured activity includes both cardiovascular and strength exercise or disease-specific rehabilitative exercises); (2) social or individual context; and (3) instruction (know-how) or information (know-why). Notably, some of the cancer survivors assumed that technology would reduce their ability to fulfill their needs (eg, enough social interaction or having a good experience). In relation to preferences, the context is important (eg, time and place; fitness center, rehabilitation center, or at home; summer or winter). Furthermore, the results indicate that those scoring lower on health technology readiness prefer activities that have a social component, whereas those scoring higher prefer to participate in individual activities (fitness center, use apps) and seek an understanding of why they should exercise and receive personalized support. We were able to identify information that can help providers offer stratified services that take the individual’s different needs and preferences into consideration. The identified preferences provide new insight that complements the cancer survivors’ readiness level and may also help designers, service providers, and caregivers to provide solutions that increase receptiveness toward technology-assisted physical activity.

### Comparison With Other Studies

Recently, 3 studies explored health technology in relation to physical activity in cancer survivors [24,25,27]. Nielsen et al [27] found that women with breast cancer undergoing chemotherapy were enthusiastic about a smartphone application designed to help them self-monitor activity between coaching sessions, and that they wanted to include family and friends and have access to a personalized tailored application. In alignment with this, Puszkiewicz et al [25] found that colorectal, breast, and prostate cancer survivors who used a generic app to support physical activity during a six-week intervention felt that an application for cancer survivors should be tailored to the individual’s lifestyle and enable social support from friends and family. Robertson et al [24] added another dimension to these findings with a focus on tools for personal goal setting to support personally held priorities and values. In all 3 studies, recruitment appeared to favor a selection of interviewees familiar with technology and who appeared to be more homogenous than our population. Our approach of also including cancer survivors who were less digitally ready may provide us with broader insight regarding the various needs and preferences of cancer survivors. Although all our interviewees would benefit from stratification, non–digital solutions, varying degrees of social interaction, and incidental physical activity also play a role, just as it must be kept in mind that not everyone wanted to know why they should be physically active.

### Implications

A recent synthesis of the acceptability and engagement of web-based interventions for cancer survivors suggests that future work should also involve identifying the optimal stage of cancer survivorship to facilitate intervention delivery [45]. We suggest, in contrast, that technology be introduced to cancer survivors at any stage of the cancer trajectory but that health professionals must take into account the identified patient preferences together with their readiness level. This could be done by either assisting cancer survivors in selecting applications from a library based on a declaration of specific content as described by Short et al [26] or by designing applications [25] that can adapt to the cancer survivor’s preferences and readiness level. By hosting a variety of existing applications [46] selected to cover the various readiness levels and preferences, more advanced digital platforms could also serve to aid adoption of technology at any stage of the cancer trajectory. Some platform applications should support social interaction, while others should aim to provide an underlying understanding of the activities and how they contribute to the cancer survivors’ health, increasing their ability to manage their condition. For those high in self-management, more individualized applications should be available and potentially incorporate personal coaching. For those not interested in structured programs, incentives to increase incidental activity should be provided to enable cancer survivors to create a palette of services better suited to their individual needs.

The data presented here and in our previous studies relates to a broad spectrum of cancer survivors, including cancers related to a high sociodemographic status (eg, breast and prostate cancer) and to a low status (eg, lung cancer and some head and neck cancers) [47]. The present population also consisted of individuals with one or more long-term conditions. Although cancer survivors may have specific needs in relation to treatment, such as prevention of lymphedema, the proposed model for digital technology–assisted physical activity during rehabilitation is likely to meet their personal needs and competences in other settings. Currently, we are exploring the READHY profile of other groups (eg, patients with type 2 diabetes mellitus and cardiovascular diseases). Qualitative studies with additional samples and other patient groups may contribute to identifying whether or not our findings are generalizable or specific to our sample.

### Strengths and Limitations

A strength of this study is that recruitment was based on stratification into READHY profiles, ensuring a broad representation of individuals regarding eHealth literacy and self-management. In addition, participants represented several different cancer types, which increases the generalizability of the findings. This study also has limitations. For example, analysis of the interview content originally applied deductive coding, but in the iterative process of creating the subcategories, we came closer to an abductive [40] workflow less influenced by the initial READHY structure. This may introduce bias but may also be a strength as the abductive approach offers an opportunity to interpret our findings in a new way in the process of analyzing the data. We fully acknowledge that other factors also need to be addressed when discussing the likelihood of adoption, such as perceived ease of use and usefulness, usability, and the user experience. We did not recruit many male participants, and we did not recruit as many participants from each profile as planned (3 per profile). This may have caused important perspectives on incidental and structured technology-assisted physical activity to be overlooked. In our previous quantitative study, we showed that age and education are associated with readiness for technology, while sex is not. The age and educational level of the interviewees may therefore influence perspectives. By discussing our results in relation to the READHY profile, we hope to have adjusted for this to a certain extent. Although the interviewer was not blinded to the profile of the interviewees, keeping an open mind was a strict priority.

### Conclusion

Combining digital technology based on the cancer survivors’ needs, preferences, and readiness with capacity building of the workforce will aid in tailoring digital solutions to suit individuals able and receptive to using them but also to those reluctant to do so. This may contribute to expanding the number of cancer survivors able to take advantage of technology-assisted services and to allocating more in-person resources to those unable to take advantage of technology. For individuals who are unreceptive to using technology, the awareness of the variations in preferences regarding incidental and structured physical activity, social interaction, and the need to receive an explanation about why exercises are important may also help motivate more cancer survivors to participate in physical activity during rehabilitation without digital support.

## Appendix

Multimedia Appendix 1 Interview guide.

Multimedia Appendix 2 Codebook.

Multimedia Appendix 3 Noninformant characteristics.

## Abbreviations

READHY: Readiness and Enablement Index for Health Technology
HeiQ: Health Education Impact Questionnaire
HLQ: Health Literacy Questionnaire
eHLQ: eHealth Literacy Questionnaire
