# Supplementary material for: Cancer Survivors’ Receptiveness to Digital Technology–Supported Physical Rehabilitation and the Implications for Design: Qualitative Study
Source: J Med Internet Res. 2020 Aug 5;22(8):e15335. doi: 10.2196/15335 (PMC7439140; doi:10.2196/15335)
Supplement: Multimedia Appendix 1 [file jmir_v22i8e15335_app1.docx]

**Multimedia Appendix 1. Interview Guide**

What has your daily life been like since being referred to the center?

Prompts

- Have you been able to do what you usually do?
- Exercise – did you do it before, do you do it less/more?
- Exercise at the center?
- Work?
- Socially/family/friends?

Can you tell me something about the support/help you get from those around you?

Prompts

- Family?
- Friends?
- Health professionals?
- Has anyone been difficult to talk to?
- Other networks – social media (Facebook)?
- Support using technology?

Technology means equipment/devices, e.g., smartphones, computers, and sports watches. Anything that can be used to show, register, or handle data digitally, as well as digital health systems such as the national Danish health portal sundhed.dk.

Can you give me some examples of how you use technology in connection with your health?

- - If not, do you use technology for other things?
  - Why/why not?

Can you think of technology that is nice to use, e.g., during leisure time or work)?

- - Why is it nice to use?

What challenges do you experience when using technology?

- - Is there something that could help you with, e.g., apps and websites?

What are your thoughts on using an app or the internet in connection with exercise/diet/treatment?

What should an app/program be able to do, if it were to make sense for you to use during rehabilitation?
