# Supplementary material for: Cancer Survivors’ Receptiveness to Digital Technology–Supported Physical Rehabilitation and the Implications for Design: Qualitative Study
Source: J Med Internet Res. 2020 Aug 5;22(8):e15335. doi: 10.2196/15335 (PMC7439140; doi:10.2196/15335)
Supplement: Multimedia Appendix 2 [file jmir_v22i8e15335_app2.docx]

**Multimedia Appendix 2. Codebook**

| **Concept** | **Dimension/code** | **Short description** |
| --- | --- | --- |
| **Self-management** | HeiQ3 Self-monitoring and insight | Ability to monitor condition, realistic about disease-related limitations |
|  | HeiQ4 Constructive attitudes and approaches | Will not let the disease control their lives |
|  | HeiQ5 Skills and technique acquisition | Ability to handle disease-related symptoms (including use of aids) |
|  | HeiQ8 Emotional distress | Emotional responses to illness, including anxiety, anger, and depression |
| **Support** | HLQ1 Feeling understood and supported by healthcare providers | Ability to talk to/engage with and trust health professionals |
|  | HLQ4 Social support for health | Have the support they want or need for health |
| **eHealth literacy** | eHLQ1 Using technology to process health information | Use technology in connection with health/disease, understand context-specific language, and critically appraise information |
|  | eHLQ2 Understanding of health concepts and language | Ability to understand test results, basic physiological functions, aware of risk factors and how to avoid them |
|  | eHLQ3 Ability to actively engage with digital services | Comfortable using digital services for handling information |
|  | eHLQ4 Feel safe and in control | Personal data safely stored and accessed only by people it is relevant to |
|  | eHLQ5 Motivated to engage with digital services | Engaging with digital services useful in managing health |
|  | eHLQ6 Access to digital services that work | Digital health data and services that work and are available when needed |
|  | eHLQ7 Digital services that suit individual needs | Access to digital services that suit users’ specific needs and preferences |
| **Digitally-assisted physical activity** | Attitude towards technology-assisted physical activity | Attitude toward technology-assisted physical activity |
|  | Technology preferences | Preferences or needs concerning a technology |
| **Attitude towards physical activity** | Attitude towards physical activity | Thoughts on exercise and physical activity; what they do |

HeiQ: Health Education Impact Questionnaire
HLQ: Health Literacy Questionnaire
eHLQ: eHealth Literacy Questionnaire.
