# Supplementary material for: Cancer Survivors’ Receptiveness to Digital Technology–Supported Physical Rehabilitation and the Implications for Design: Qualitative Study
Source: J Med Internet Res. 2020 Aug 5;22(8):e15335. doi: 10.2196/15335 (PMC7439140; doi:10.2196/15335)
Supplement: Multimedia Appendix 3 [file jmir_v22i8e15335_app3.docx]

**Multimedia Appendix 3. Non-informant Characteristics**

| ID | Sex | Age | READHY profile^a^ | Physical activity digital technology usage^b^ | Receptive^c^ | Technology ownership^c^ | | | Usage^c^ | Purpose of using technology^c^ | | | | |
| --- | --- | --- | --- | --- | --- | --- | --- | --- | --- | --- | --- | --- | --- | --- |
|  |  |  |  |  |  | Smartphone | Tablet | Computer |  | Work | Information seeking | Communication (family/friends) | Practicalities | Exercise |
|  | | | | | | | | | | | | | | |
| - | M | 60 | 4 | - | Yes | X | X | X | Several times daily | X |  | X | X | X |
| - | M | 28 | 1 | - | Yes | X | X | X | Several times daily |  | X | X |  |  |
| - | F | 41 | 1 | - | No | X | X | X | Several times daily | X | X | X |  |  |
| - | F | 58 | 1 | - | No |  |  |  | Few times a month |  | X |  | X |  |
| - | M | 47 | 2 | - | Yes | X | X | X | Several times daily | X | X | X | X |  |
| - | M | 72 | 2 | - | Yes |  | X |  | Several times daily |  | X | X |  |  |
| - | M | 69 | 4 | - | Yes | X | X | X | Several times daily | X | X | X | X | X |
| - | F | 64 | 1 | - | Yes | X | X | X | Several times daily |  | X | X |  |  |
| - | M | 70 | 1 | - | No |  |  |  | Never/almost never |  |  |  |  |  |
| - | M | 58 | 1 | - | No | X |  | X | Few times a week | X |  |  |  |  |
| - | M | 66 | 1 | - | No |  | X |  | Several times a week |  |  | X |  |  |
| - | F | 39 | 4 | - | Yes | X | X | X | Several times daily |  | X | X | X |  |

^a^ READHY: Readiness and Enablement Index for Health Technology. ^b^ Physical activity digital technology usage is based on the informant statements. ^c^ Based on questionnaire data reported in [1].

1. Rossen S, Kayser L, Vibe-Petersen J, Ried-Larsen M, Christensen JF. Technology in exercise-based cancer rehabilitation: a cross-sectional study of receptiveness and readiness for e-Health utilization in Danish cancer rehabilitation. Acta Oncol Stockh Swed 2019 Jan 30;1–9. PMID:30698060
